# Codeine-Associated Secondary Adrenal Insufficiency: A Case Report

**DOI:** 10.7759/cureus.118882

**Published:** 2026-10-31

**Authors:** Eleesha A Varghese, Nathan T Zachariah, Sunil Zachariah

**Affiliations:** 1 General Medicine, Kingston Hospital, London, GBR; 2 Internal Medicine, Dr. B.R. Ambedkar Medical College, Bangalore, IND; 3 Diabetes and Endocrinology, Surrey and Sussex Healthcare NHS Trust, Surrey, GBR

**Keywords:** acth, adrenal crisis case report, adrenal insufficiency in elderly, codeine, low cortisol, morphine use, opioids use

## Abstract

We report the case of a 47-year-old woman who presented with a two-month history of significant lethargy, dizziness, and weakness. Blood tests showed a significantly low 9:00 am cortisol level of 52 nmol/L (normal: >275 nmol/L), an abnormal Synacthen test, and low adrenocorticotropic hormone (ACTH) levels. The remaining pituitary function tests and MRI of the pituitary and hypothalamus were normal. Interestingly, she had been taking the maximum licensed dose of codeine for back pain for 12 weeks before the blood tests. Adrenal insufficiency was thought to be induced by codeine, and the medication was stopped immediately. Her symptoms resolved spontaneously without steroid replacement, and repeat investigations six months later showed full recovery of adrenal function. Opioids suppress cortisol secretion, mainly through negative feedback on the hypothalamus and pituitary gland. The literature suggests that opioids also interact with other endocrine functions. Although hypogonadotropic hypogonadism is the most reported endocrine effect, opioids also exert inhibitory effects on the corticotropic axis.

## Introduction

Opioids are widely prescribed analgesics in the developed world for the management of acute and chronic pain and form part of the WHO analgesic ladder [1-3]. Evidence shows a 35% increase in opioid prescribing in England between 1998 and 2018 [4]. Opioids are increasingly used in the management of chronic cancer- and non-cancer-related pain, as well as moderate to severe acute pain [5]. Given their widespread use, prescribers are often aware of common adverse effects such as constipation, nausea, and sedation, as well as the potential for addiction and tolerance [6-8]. In chronic opioid users, hypogonadotropic hypogonadism is well reported, with frequency as high as 89% in males and 67% in females [9-11]. However, opioid-induced adrenal insufficiency (OIAI) remains highly underrecognized, possibly due to its vague symptomatology and a lack of awareness among clinicians [6,12,13]. Studies have reported an estimated average diagnostic delay of six months, with approximately 20% of patients waiting more than five years to receive a diagnosis and nearly 70% initially receiving an incorrect diagnosis [14].

The mechanism of OIAI is thought to be due to suppression of the hypothalamus-pituitary-adrenal (HPA) axis [15,16]. The HPA axis is a neuroendocrine pathway that helps regulate physiological homeostasis through a negative feedback loop mechanism [17]. Hence, OIAI is classified as secondary or central adrenal insufficiency, as its underlying mechanism is thought to involve inadequate stimulation of the adrenal glands by pituitary adrenocorticotropic hormone (ACTH). This contrasts with primary adrenal insufficiency, which results from intrinsic dysfunction of the adrenal glands, impairing their ability to produce corticosteroids [18]. The reported prevalence of OIAI varies considerably, approximately 8.3%-29% in individual contemporary clinical studies [19-22]. A systematic review and meta-analysis reported a weighted mean prevalence of approximately 15% [23]. The heterogeneity largely reflects differences in patient populations, opioid exposure, cortisol assays, and diagnostic thresholds. Prompt recognition of OIAI is crucial, as delay in diagnosis can lead to life-threatening complications such as adrenal crisis [24].

This case report aims to raise awareness among clinicians of adrenal insufficiency as an important differential diagnosis in patients receiving opioid therapy who present with non-specific symptoms such as lethargy and dizziness. It highlights the importance of timely investigation and appropriate management, including opioid withdrawal and glucocorticoid replacement where indicated, to prevent delayed or missed diagnosis.

## Case presentation

A 47-year-old woman presented in August 2025 with a two-month history of increased tiredness and lethargy, postural dizziness, and weakness. Her only past medical history was a left-sided hemithyroidectomy in 2021 for a large benign thyroid nodule, and she was taking a stable dose of levothyroxine. On reviewing her previous records, it was interesting to note that blood tests performed in January 2025 showed a normal 9:00 am cortisol level of 482 nmol/L (normal: >275 nmol/L) and a normal TSH level of 1.68 mIU/L (normal range: 0.27-4.20 mIU/L). There was no family history of autoimmune disorders. Her menstrual periods were regular, and she had no history of headache, weight loss, or visual symptoms.

Clinical examination revealed a blood pressure of 108/62 mmHg, with a postural drop of 12 mmHg. There was no evidence of palmar or buccal pigmentation.

Blood tests (Table 1) showed a significantly low 9:00 am cortisol level of 52 nmol/L, with normal urea and electrolyte levels. The remainder of the pituitary function tests were normal. She subsequently underwent an urgent short Synacthen test, which was abnormal, with a zero-minute cortisol level of 79 nmol/L (normal: >100 nmol/L), a 30-minute cortisol level of 195 nmol/L (normal: >430 nmol/L or an increase of 200 nmol/L from the zero-minute value), and a 60-minute cortisol level of 288 nmol/L. Her ACTH level was low at 2 ng/L (normal range: 10-50 ng/L), and the adrenal cortex antibody test was negative.

**Table 1:** Blood test results ACTH: Adrenocorticotropic hormone; TSH: Thyroid-stimulating hormone.

Blood test	Normal value	Baseline (January 2025)	2 months after symptom onset (August 2025)	6 months following stopping codeine
9 am cortisol	>275 nmol/L	482 nmol/L	52 nmol/L	380 nmol/L
ACTH	10-50 ng/L	-	2 ng/L	22 ng/L
TSH	0.27-4.20 mIU/L	1.68 mIU/L	-	-
Adrenal cortex antibody	-	-	Negative	-

There was no history of previous use of steroid tablets, creams, inhalers, or injections. Interestingly, on further history-taking, she reported taking the maximum licensed dose of codeine phosphate (60 mg four times a day) for back pain following a mechanical injury for three months before the blood tests. She was not taking any other opiates. An MRI scan of the pituitary and hypothalamus with contrast was normal, and the patient was diagnosed with opiate-induced isolated ACTH deficiency.

Regular hydrocortisone replacement therapy was advised; however, the patient was reluctant to take it regularly. A supply for emergency use and an emergency steroid injection kit were therefore provided, but she did not use them. She stopped taking codeine immediately, and her symptoms began to resolve within a few weeks. Repeat blood tests (Table 2) performed six months later showed full recovery of adrenal function, with a 9:00 am cortisol level of 380 nmol/L and a short Synacthen test showing a zero-minute cortisol of 282 nmol/L, a 30-minute cortisol of 444 nmol/L, and a 60-minute cortisol of 620 nmol/L, with a normal ACTH level of 22 ng/L.

**Table 2:** Short Synacthen test ACTH: Adrenocorticotropic hormone.

Short Synacthen test (250 mcg intravenous synthetic ACTH)	Normal value	Initial results	Repeat test 6 months after stopping codeine
Cortisol level at 0 minutes	>100 nmol/L	79 nmol/L	282 nmol/L
Cortisol level at 30 minutes	>430 nmol/L or increase of 200 nmol/L from zero-minute value	195 nmol/L	444 nmol/L
Cortisol level at 60 minutes	-	288 nmol/L	620 nmol/L

Despite being offered hydrocortisone replacement therapy, the patient did not take it. Adrenal function recovered fully following discontinuation of codeine alone, as demonstrated by the repeat short Synacthen test. Although a rechallenge was not performed, application of the Naranjo Adverse Drug Reaction Probability Scale indicated a probable causal relationship between codeine therapy and secondary adrenal insufficiency (Figure 1).

**Figure 1:**
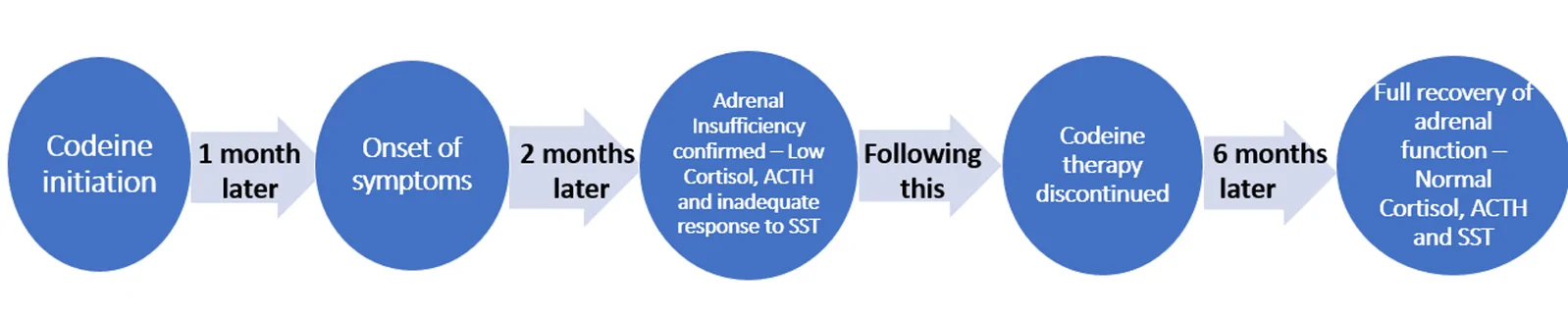
Timeline of events ACTH: Adrenocorticotropic hormone; SST: Short Synacthen test.

## Discussion

OIAI is a recognized cause of adrenal dysfunction; however, it remains underdiagnosed, with only approximately 10% of affected patients being appropriately diagnosed [25]. Although OIAI has a notable prevalence in the current opioid epidemic and can occur with any opioid, there remains a paucity of research and clinical trials in this area.

The HPA axis is a feedback loop involving the hypothalamus, anterior pituitary, and adrenal glands that regulates hormonal balance, controls the body's physiological response to stress, and influences metabolism and autonomic function [17]. Evidence suggests that short-term and long-term use of opiates can suppress the HPA axis due to their inhibitory effects on ACTH secretion [6,26,27]. Opioids exert their effects through G protein-coupled receptors (μ, δ, and κ receptors), which are present throughout the body, specifically in the hypothalamus and pituitary gland [28]. Although the exact mechanism is not fully understood, HPA dysfunction can trigger variations in neuropeptide and neurotransmitter synthesis, resulting in physiological imbalances and disruptions [17,29]. This unfolds as secondary or central adrenal insufficiency rather than primary adrenal insufficiency, in which the adrenal glands are directly affected by disease [26] (Figure 2).

**Figure 2:**
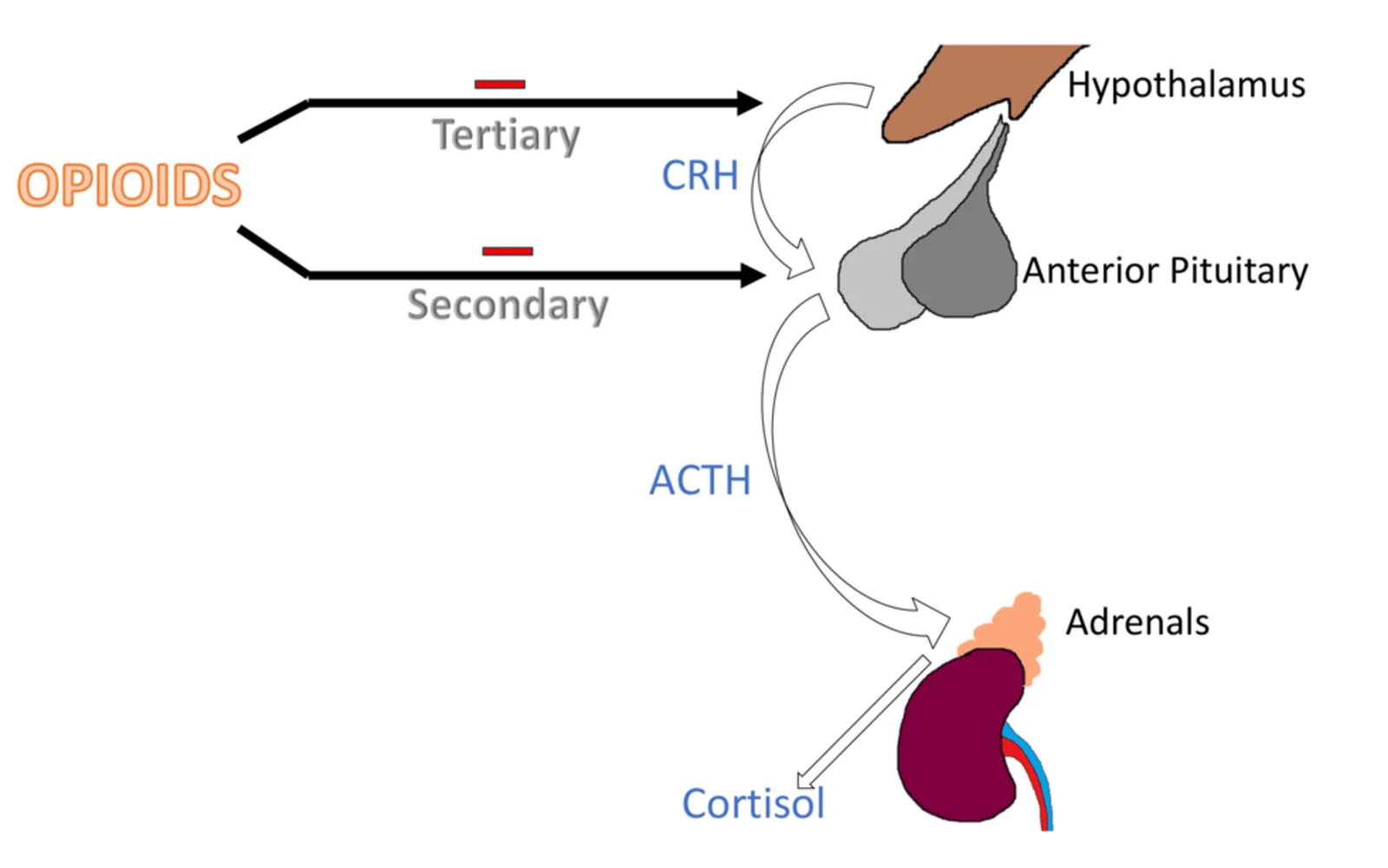
Inhibitory action of opioids on hypothalamus-pituitary-adrenal axis ACTH: Adrenocorticotropic hormone; CRH: Corticotropin-releasing hormone.

There is no conclusive evidence demonstrating the risk associated with different opioid formulations, doses, and routes of administration in the development of OIAI [22]. In general, the consensus is that patients taking higher doses of morphine milligram equivalents (MME) per day have higher chances of developing OIAI, and the strongest risk factor may be long-term opioid exposure [19,20]. Some studies show that patients with higher cumulative opioid exposure are at increased risk of developing OIAI [20].

Cortisol is the principal serum glucocorticoid secreted by the adrenal glands and follows a circadian pattern, peaking approximately 30 minutes to 2 hours after waking. Cortisol secretion also increases in response to physical and emotional stress [30]. The clinical features of OIAI are explained by the relative or absolute deficiency of cortisol and include lethargy, unintentional weight loss, dizziness due to postural hypotension, muscle aches, abdominal pain, and nausea or vomiting [31]. This makes the diagnosis challenging, as these symptoms are nonspecific and may be misinterpreted as being part of an underlying chronic pain syndrome [32,33].

OIAI is fundamentally related to low cortisol levels, particularly affecting the peak cortisol pattern within the circadian rhythm [28]. Hence, the European Society of Endocrinology recommends morning cortisol measurement as the first step in the diagnosis of adrenal insufficiency. In cases where the results are inconclusive, measurement of ACTH and a synthetic corticotropin stimulation test (CST) are recommended [34]. In patients with OIAI, the initial expected findings include a low morning cortisol level with a low or inappropriately normal ACTH concentration. Corticotropin stimulates the release of cortisol; however, this response depends on the functional capacity of the adrenal cortex. In OIAI, the expected finding is therefore a failure to mount an adequate cortisol response to CST. As the CST is dependent on the adrenal cortex, adrenal atrophy, either due to primary adrenal pathology or prolonged absence of corticotropin stimulation, can result in an abnormal response. The response to CST may be inadequate depending on the degree of background corticotropin stimulation, which is, in turn, influenced by the dose and duration of opioid use, as well as individual susceptibility.

In such situations, administration of a supraphysiological dose of synthetic corticotropin may stimulate the adrenal cortex sufficiently to produce a cortisol response, which can aid in the diagnosis of OIAI. Therefore, the results of CST should be interpreted with caution, as there is evidence that OIAI can occur even in patients with a normal CST. Furthermore, there is currently no consensus regarding the cut-off value for CST in the diagnosis of OIAI [6]. The insulin tolerance test is considered the gold-standard diagnostic test for OIAI; however, its limited availability, cost, and the need for additional precautions in patients with ischemic heart disease or seizures limit its use [20,22]. Although there is no consensus regarding the optimal diagnostic approach, the most commonly used strategy is a combination of morning cortisol, ACTH, and CST [35]. In addition, a low dehydroepiandrosterone sulfate (DHEAS) level, an adrenal androgen released in response to corticotropin stimulation, may occur concurrently with low ACTH availability and can provide further support for the diagnosis [36].

A low ACTH level is suggestive of central or secondary adrenal insufficiency; hence, other confounding differentials, such as exogenous glucocorticoid administration, pituitary tumors, head trauma, infiltrative disease, and apoplexy, should be ruled out before diagnosing OIAI [37].

There are two main approaches to the treatment of OIAI: the first is discontinuation of opioids, and the second is treatment with exogenous glucocorticoids. As demonstrated in the case described here, there is evidence that cessation of opioids can result in complete reversal of OIAI without the need for glucocorticoid supplementation [15,20,33,38]. The time to full recovery following opioid discontinuation may vary [6]. Care should be taken to gradually wean opioids in patients receiving long-term therapy to avoid withdrawal symptoms associated with sudden cessation [39,40]. If complete cessation cannot be achieved, the lowest possible opioid dose should be prescribed [41].

The alternative approach is to prescribe exogenous glucocorticoids, most commonly hydrocortisone, in two or three divided doses, with the majority of the dose given in the morning to mimic the physiological pattern of cortisol release. Hydrocortisone is preferred over other glucocorticoids, such as prednisolone and dexamethasone, which have longer half-lives and may improve patient compliance but can increase the risk of suppression of endogenous cortisol production and side effects associated with excessive steroid exposure [42]. As the underlying mechanism of OIAI is suppression of the HPA axis rather than destruction of the adrenal cortex, it does not cause mineralocorticoid deficiency and therefore does not require aldosterone supplementation [6]. It is advised that adrenal function should be assessed periodically in patients receiving glucocorticoid replacement, particularly following the tapering or discontinuation of opioids, as adrenal function may recover and it may subsequently be possible to discontinue steroid supplementation [25].

Patients with OIAI are at risk of adrenal crisis, which is a medical emergency. They may present with nausea, vomiting, hypotension, or even collapse and require urgent treatment with intravenous fluid resuscitation and intravenous glucocorticoids [43]. It is crucial to counsel patients regarding "sick day rules," including increasing the steroid dose by two- to three-fold during intercurrent illness and periods of physiological stress, such as trauma or surgery, to prevent adrenal crisis. Where appropriate, patients should also be provided with a supply of intramuscular glucocorticoids for use at home in an emergency [44,45]. Wearing a medical alert bracelet or necklace can also be particularly helpful in ensuring that healthcare responders are aware of the diagnosis during an emergency [44]. Counselling patients about these potential endocrine complications should be considered prior to initiating opioid therapy [6,46]. Previously published case reports and abstracts have documented adrenal insufficiency associated with various opioids and different glucocorticoid replacement approaches (Table 3).

**Table 3:** Different types of opiates causing adrenal insufficiency in previously published case reports/abstracts

Year	Title	Opioid Used	Management	Reference
2026	An Unrecognized Cause of Chronic Diarrhea: Opioid-Induced Adrenal Insufficiency – A Case Report	Buprenorphine	Hydrocortisone replacement	Donkor et al. [47]
2026	When Dysautonomia Is Not the Answer: A Case of Missed Opioid-Induced Adrenal Insufficiency	Oxycodone	Prednisolone replacement	Afzal et al. [48]
2026	Opioid-Induced Adrenal Insufficiency With Concurrent Opioid-Induced Esophageal Dysfunction Presenting With Achalasia-Like Features	Methadone	Hydrocortisone replacement	Owusu-Ansah et al. [49]
2025	Severe Hypoglycaemia Secondary to Chronic Opioid-Induced Hypothalamic–Pituitary–Adrenal Axis Suppression: An Under-Recognised Phenomenon	Codeine and Oxycodone	Hydrocortisone replacement	Do et al. [50]
2025	A Rare Case of Codeine-Induced Secondary Adrenal Insufficiency With Hyponatremia Worsened by Hydrochlorothiazide: A Case Report and Review of Literature	Codeine	Hydrocortisone replacement	Kermaj et al. [13]
2024	P-60 Opioid-Induced Secondary Adrenal Insufficiency: A Case Report	Methadone	Emergency hydrocortisone therapy	Rocha et al. [51]
2024	Opioid-Induced Adrenal Insufficiency in a Patient Treated With Buprenorphine-Naloxone: A Case Report	Buprenorphine	Hydrocortisone replacement	Ahmed et al. [52]
2024	Opioid-Induced Adrenal Insufficiency in a Patient Presenting With Acute Adrenal Crisis	Heroin	Hydrocortisone replacement	Najjar et al. [53]
2024	Opioid-Induced Adrenal Insufficiency	Methadone	Steroid replacement	Rice and Yoshida [54]
2023	THU624 Opioid-Induced Adrenal Insufficiency (OIAI) Presenting With Altered Mental Status	Buprenorphine	Hydrocortisone replacement	Bondarenko et al. [55]
2023	Long-Term Opiate Therapy-Induced Secondary Adrenal Insufficiency: A Distinct Differential Diagnosis That Should Be Considered	Morphine	Hydrocortisone replacement	Khair [56]
2022	Opioid-Induced Adrenal Insufficiency	Oxycodone and Heroin	Hydrocortisone replacement	Bush and Barbero [57]
2022	Opioid-Induced Central Adrenal Insufficiency Presenting as Fever of Unknown Origin	Oxycodone + Roxicodone	Hydrocortisone replacement	Berardi et al. [58]
2021	Non-fasting Hypoglycemia Secondary to Opioid-Induced Adrenal Insufficiency: A Case Report	Methadone	Hydrocortisone replacement	Kubbar and Banerji [59]
2021	Opioid-Induced Secondary Adrenal Insufficiency in a Young Patient With Chronic Pancreatitis	Oxycodone	Prednisolone replacement	Majeed et al. [60]
2020	Isolated Adrenocorticotropic Hormone Deficiency Secondary to Chronic Opiate Use	Hydrocodone	Hydrocortisone replacement	Raj et al. [61]
2018	Opioid-Induced Secondary Adrenal Insufficiency	Tramadol	Prednisone replacement	Petre and Albu [62]
2017	Secondary Adrenocortical Insufficiency as a Result of Chronic Fentanyl Treatment: A Case Report	Transdermal fentanyl	Hydrocortisone replacement	Esteban-Zubero et al. [63]
2015	Opioid-Induced Secondary Adrenal Insufficiency Presenting as Hypercalcaemia	Methadone	Hydrocortisone replacement	Lee and Twigg [64]
2014	A Case of Adrenal Insufficiency Secondary to Chronic Opioid Use – Keep This Diagnosis in Mind!	Morphine	Steroid supplementation	Rabi [65]
2014	Opioid-Induced Adrenal Insufficiency in Transdermal Fentanyl Treatment: A Revisited Diagnosis in Clinical Setting	Transdermal fentanyl	Oral corticosteroid replacement	Kondo et al. [66]
2011	Tramadol-Induced Adrenal Insufficiency	Tramadol	Steroid replacement	Debono et al. [38]
2005	Chronic Fentanyl Application Induces Adrenocortical Insufficiency	Transdermal fentanyl	Hydrocortisone replacement	Oltmanns et al. [15]

## Conclusions

Opioids are widely used as a mainstay of pain management in the developed world. Although their common side effects are well recognized, opioid-induced endocrinopathies remain a relatively underappreciated complication. Studies indicate significant diagnostic delays and frequent initial misdiagnosis. The lack of awareness of these conditions, along with the absence of consensus regarding their diagnosis and treatment, contributes to these delays. OIAI is associated with increased morbidity and, if undiagnosed, can precipitate life-threatening emergencies such as adrenal crisis. Long-term opioid use can cause hypogonadism and adrenal insufficiency in both men and women. Therefore, appropriate counselling regarding these potential complications may be considered before initiating opioid therapy. Clinicians should consider taking a comprehensive drug history that extends beyond oral, topical, and inhalational steroid use to specifically enquire about opioid intake in cases of suspected adrenal insufficiency.

This case highlights the need to increase clinicians’ knowledge and awareness of opioid-induced endocrine dysfunction, including adrenal insufficiency secondary to ACTH suppression. This is particularly relevant given the increasing use of opioids worldwide.
